# Ascertaining the Kawasaki Disease–Coronavirus Disease 2019 Linkage in a Japanese Administrative Claims Database

**DOI:** 10.1111/ped.70420

**Published:** 2026-04-28

**Authors:** Tomoki Mizuno, Jun Suzuki, Shota Takahashi, Haruka Imai, Hideya Itagaki, Tomohiro Akaba, Makiko Yoshida, Shiro Endo

**Affiliations:** ^1^ Division of Infectious Diseases Tohoku Medical and Pharmaceutical University Hospital Sendai Miyagi Japan; ^2^ Department of Infection Prevention and Control Tohoku Medical and Pharmaceutical University Hospital Sendai Miyagi Japan; ^3^ Division of Infectious Diseases and Infection Control, Faculty of Medicine Tohoku Medical and Pharmaceutical University Sendai Miyagi Japan; ^4^ Division of Infectious Diseases and Infection Control, Department of Social and Community Medicine, Graduate School of Medicine Tohoku Medical and Pharmaceutical University Sendai Miyagi Japan; ^5^ Department of Respiratory Medicine Tokyo Women's Medical University School of Medicine Tokyo Japan; ^6^ Division of the Crisis Management Network for Infectious Diseases Tohoku Medical and Pharmaceutical University Sendai Miyagi Japan

**Keywords:** COVID‐19, multisystem inflammatory syndrome in children, SARS‐CoV‐2, severe acute respiratory syndrome coronavirus 2

## Abstract

**Background:**

Despite the potential association between Kawasaki Disease (KD) and coronavirus disease 2019 (COVID‐19), real‐world clinical associations remain unclear. We aimed to investigate the association between KD and COVID‐19 using a Japanese claims database.

**Methods:**

Using a Japanese claims database, we identified patients diagnosed with COVID‐19 between January 1, 2020, and December 31, 2022 and conducted the following two analyses: a nested case–control study wherein each KD case was matched 1:4 to controls (without KD), and conditional logistic regression analyses were used to calculate odds ratios (ORs) for COVID‐19 exposure in the previous 1–30, 31–60, and 61–90 days. In the self‐controlled case‐series (SCCS) analysis, the observation comprised the 120 days before and after COVID‐19 diagnosis (Day 0). We calculated the incidence rate ratio (IRR) of three risk periods (Days 1–30, 31–60, 61–90) after Day 0 compared with the control periods.

**Results:**

Nested case–control analysis matched 134 cases with 536 controls. COVID‐19 exposure was identified in 26.1% (35/134) of cases and 7.3% (39/536) of controls during the previous 1–30 days (adjusted OR 7.8, 95% CI 3.2–19), and in 18.7% (25/134) and 8.8% (47/536) during the previous 31–60 days (adjusted OR 3.6, 95% CI 2.0–6.5). SCCS analysis included 124 patients, revealing elevated IRRs on Days 1–30 (95% CI: 11 [6.0–20]), and 31–60 (6.9 [3.6–13]) after COVID‐19 diagnosis.

**Conclusion:**

The present study found that COVID‐19 was associated with the development of KD.

## 1 Introduction

Coronavirus disease 2019 (COVID‐19), caused by a severe acute respiratory syndrome coronavirus 2 (SARS‐CoV‐2) infection, remains a significant health problem worldwide [1]. Despite tending to have mild symptoms without severe or critically ill status [2], children, even after recovering from COVID‐19, have an increased risk of developing different health problems [3].

Kawasaki disease (KD), which is frequently reported in Northeast Asian countries, including Japan, is a pediatric vasculitis that potentially causes coronary artery aneurysms [4]. Despite its unknown etiology, various theories have been proposed based upon pathologic, epidemiologic, and demographic data. One of these theories is that KD is triggered by infectious etiology, such as respiratory syncytial virus and enteroviruses [5, 6, 7]. After the initiation of COVID‐19 special mitigation measures, the cases of KD declined, possibly because of public health measures, such as masking and social distancing. This fact corroborates the infectious etiology of KD [8, 9]. On the other hand, several retrospective studies indicate that KD may be triggered by SARS‐CoV‐2, one of the respiratory viruses [10, 11]. However, its epidemiological features in Japan remain unclear.

Therefore, we investigated the epidemiological relationship between COVID‐19 and KD, using a large‐scale medical claims database.

## 2 Methods

### 2.1 Database Information

We used the Japanese inpatient and outpatient administrative claims database of Medical Data Vision Co. Ltd. (Tokyo, Japan) which contains claims data for approximately 40 million patients from 28% of 1761 acute care hospitals [12]. The database includes patient demographics (age, sex, hospital scale, and diagnoses coded with the International Classification of Diseases, 10th Revision [ICD‐10]), hospitalization summary (admission and discharge dates, comorbidities, and outcomes), and medical procedures and drugs coded using Japanese original codes.

### 2.2 Study Population

We identified patients ≤ 18 years of age diagnosed with COVID‐19 (ICD‐10 codes U071 and U072) between January 1, 2020, and December 31, 2022. The day that COVID‐19 was diagnosed was defined as the day that patients were coded with U071 or U072 and received procedure codes. Using the ICD‐10 code M303 and history of the specific therapy (immunoglobulin or aspirin, procedure codes are listed in Table S1), we identified patients with a first history of KD during the study period, and the KD diagnosis day was defined as the day when these codes were recorded and the specific therapy was initiated. Comorbid conditions were defined based on the Charlson Comorbidity Index score [13, 14].

### 2.3 Nested Case–Control Study

In the study population (patients with COVID‐19), we identified patients with KD (the outcome of interest) as “cases” whereas “controls” were defined as patients who had no history of KD during the study period. For each case, we then randomly selected four controls selected on the same date as the case's hospitalization or diagnosis with KD. The cases and controls were 1:4 matched based on age, sex, inpatient or outpatient status, and the Charlson's Comorbidity Index. We excluded cases with less than 4 matched controls. We used conditional logistic regression analyses to estimate the odds ratios (ORs) for a COVID‐19 diagnosis (analyzed separately for 1–30, 31–60, and 61–90 days before the COVID‐19 diagnosis day) in matched patients with and without KD.

### 2.4 Self‐Controlled Case Series and Statistical Analysis

To investigate the relationship between a transient exposure (SARS‐CoV‐2 infection) and an acute outcome event (KD onset), we calculated the incidence rate ratio (IRR) using a self‐controlled case‐series (SCCS) method. The SCCS method compares periods of exposure and non‐exposure within the same patient using conditional Poisson regression models, inherently controlling for confounders that remain constant over time. No external controls are needed as patients serve as their own control. The SCCS method has the advantage that the effects of any time‐invariant covariates (such as sex, ethnicity, and genetic factors) are implicitly controlled, thereby minimizing confounding by indication [15].

**FIGURE 1:**
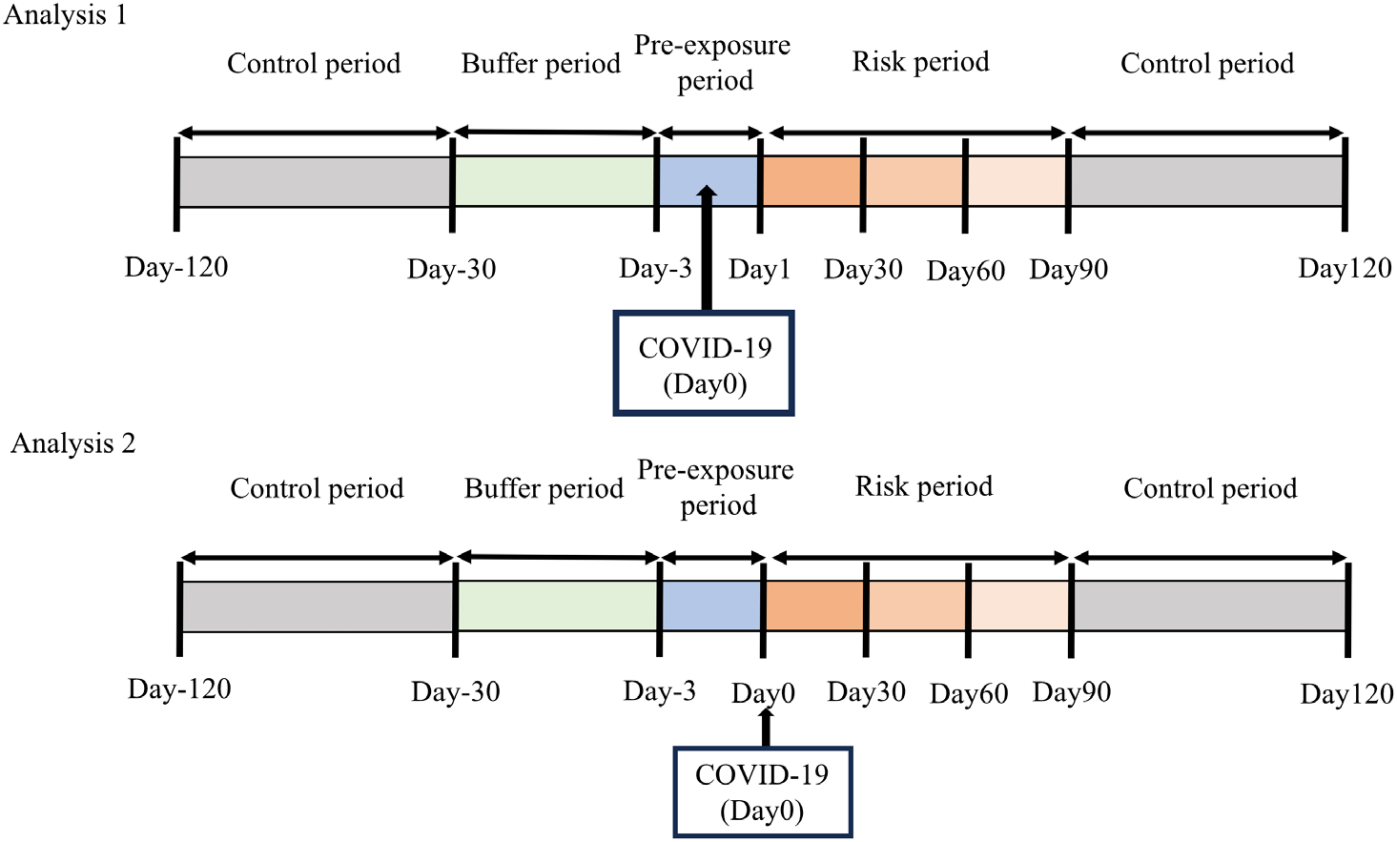
Overview of the self‐controlled case‐series study period. Analysis 1 shows the IRRs and 95% CIs of KD during the buffer period (Days −28 to −4), pre‐exposure period (Days −3 to −0), risk periods of the first and second weeks, third and fourth weeks, and fifth and sixth weeks (Days 29–42) from COVID‐19 infection, compared with the control period. Analysis 2 shows the IRRs and 95% CIs of KD during the buffer period (Days −28 to −4), pre‐exposure period (Days −3 to −1), risk periods of the first and second weeks, third and fourth weeks, and fifth and sixth weeks from COVID‐19 infection, compared with the control period. CI, confidence interval; COVID‐19, coronavirus disease 2019; IRR, incidence rate ratio; KD, Kawasaki disease.

For the SCCS method, we defined study periods based on a previous study [16]. The observation period included 120 days before and after the COVID‐19 diagnosis, with Day 0 as the diagnosis day. As shown in Figure 1, we defined the following periods, and the remaining period was defined as the control period; the buffer period (Days −30 to −4), pre‐exposure period (Days −3 to 0), first risk period (Days 1 to 30), second risk period (Days 31 to 60), and third risk period (days 61 to 90) in Analysis 1. We performed Analysis 2 because a large number of KD cases occurred on Day 0 and possibly conferred a potential test bias. In Analysis 2, we included Day 0 in the first risk period, instead of the pre‐exposure period. Thus, we calculated IRRs and 95% CIs of KD during the buffer period (Days −28 to −4), pre‐exposure period (Days −3 to −1), and the first risk period (Days 0–30), second risk period (Days 31–60), and third risk period (Days 61–90). To adjust for seasonality, we categorized seasons into four groups (Spring: March–May; Summer: June–August; Autumn: September–November; and Winter: December–February) and included them as categorical covariates in the Poisson regression model, because seasons are recognized as time‐varying confounders for KD [17, 18].

In addition, the SARS‐CoV‐2 vaccination strategies for infants aged 6 months to 4 years were started after October 25, 2022 in Japan [19]. Therefore, we defined pediatric patients ≤ 4 years who were diagnosed with COVID‐19 before October 24, 2022, as those who had not received the SARS‐CoV‐2 vaccine, and the SCCS method was performed as a sensitivity analysis.

### 2.5 Statistical Analysis

Descriptive data were reported as numbers and percentages for categorical variables and as medians and interquartile ranges for continuous variables. All analyses were performed using the “Epi”, “SCCS”, and “Survival” package in R version 4.4.1.

## 3 Results

**FIGURE 2:**
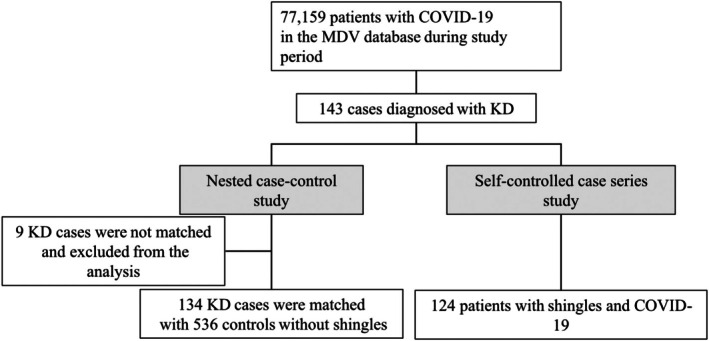
Patient‐inclusion flowchart. CI, confidence interval; COVID‐19, coronavirus disease 2019; IRR, incidence rate ratio; KD, Kawasaki disease.

In total, we identified 77,159 patients with COVID‐19. Of these patients, 143 patients were diagnosed with KD during the study period. Among 143 patients with KD, 9 patients were not matched and excluded from the nested case–control study, and 134 patients were included in the nested case–control study. On the other hand, 124 patients with KD during the 120 days before and after COVID‐19 diagnosis day were identified and included in the SCCS method (Figure 2).

In the nested case–control study, the median age was 1.0 (interquartile range [IQR], 0–3) years and 56% of both cases and controls were male (Table 1). Table 2 shows the ORs derived from conditional logistic regression analyses. In the previous 1–30 days, 26.1% (35/134) of the cases and 7.3% (39/536) of the controls were exposed to SARS‐CoV‐2 infection (adjusted OR 7.8 [95% CI 3.2–19]). In the previous 31–60 days, 18.7% (25/134) of the cases and 8.8% (47/536) of the controls were exposed to SARS‐CoV‐2 infection (adjusted OR 3.6 [95% CI 2.0–6.5]).

**TABLE 1:** Characteristics in the nested case–control study design.

Characteristics	Controls	KD cases
No. of patients	536	134
Sex, *n* (%)		
Male	300 (56.0)	75 (56.0)
Female	236 (44.0)	59 (44.0)
Age, years (median [IQR])	1.0 [0.0–3.0]	1.0 [0.0–3.0]
Charlson comorbidity index [IQR]	0 [0–1]	0 [0–1]
Diagnosis year, *n* (%)		
2020	0 (0.0)	0 (0.0)
2021	2 (0.4)	0 (0.0)
2022	534 (99.6)	134 (100)
Settings of COVID‐19 treatment, *n* (%)		
Inpatient	208 (38.8)	52 (38.8)
Outpatient	328 (61.2)	82 (61.2)

Abbreviations: COVID‐19, coronavirus disease 2019; IQR, interquartile range; KD, Kawasaki disease.

**TABLE 2:** Results of conditional logistic regression analysis in the nested case–control study.

Risk periods of COVID‐19 before the index date (days)	Controls (*n* = 536)	KD cases (*n* = 134)	Adjusted OR (95% CI)
1–30	39 (7.3)	35 (26.1)	7.8 (3.2–19)
31–60	47 (8.8)	25 (18.7)	3.6 (2.0–6.5)
61–90	35 (6.5)	4 (3.0)	0.60 (0.20–1.8)

Abbreviations: CI, confidence interval; COVID‐19, coronavirus disease 2019; KD, Kawasaki disease; OR, odds ratio.

Using the SCCS method, the median age was 1 years (interquartile range [IQR]: 0–3 years) and 57% were male (Table 3). As shown in Table 4, the IRRs of KD increased during the pre‐exposure period (68, 95% CI: 36–127), first month (11, 95% CI: 6.0–20), second month (6.9, 95% CI: 3.6–13). Another analysis showed similar results. In the sensitivity analysis, 74 patients were included and yielded similar results (Table 5).

**TABLE 3:** Characteristics identified in the SCCS design.

Characteristics	Value
No. of patients	124
Sex, *n* (%)	
Male	70 (56.5)
Female	54 (43.5)
Age, years (median [IQR])	1.0 [0.0–3.0]
Charlson comorbidity index [IQR]	0 [0–1]
Diagnosis year, *n* (%)	
2020	0 (0.0)
2021	0 (0.0)
2022	124 (100)
Settings of COVID‐19 treatment, *n* (%)	
Inpatient	65 (52.4)
Outpatient	59 (47.6)

Abbreviations: CI, confidence interval; COVID‐19, coronavirus disease 2019; IQR, interquartile range; KD, Kawasaki disease.

**TABLE 4:** IRRs of KD within the 120 days before and after a COVID‐19 diagnosis.

	Events, *n*	IRR (95% CI)
Analysis: Day 0 out of the risk period	124	
Control period	15	Reference
Buffer period: Days −30 to −4	2	0.63 (0.14–2.7)
Pre‐exposure period: Days −3 to 0	33	68 (36–127)
Risk period: first month (Days 1 to 30)	41	11 (6.0–20)
Risk period: second month (Days 31 to 60)	27	6.9 (3.6–13)
Risk period: third month (Days 61 to 90)	6	1.5 (0.58–3.9)
Analysis: Day 0 in the risk period		
Control period	15	Reference
Buffer period: Days −30 to −4	2	0.61 (0.14–2.7)
Pre‐exposure period: Days −3 to −1	2	5.4 (1.2–24)
Risk period: first month (Days 0 to 30)	72	18 (11–33)
Risk period: second month (Days 31 to 60)	27	6.9 (3.7–13)
Risk period: third month (Days 61 to 90)	6	1.5 (0.59–4.0)

Abbreviations: CI, confidence interval; COVID‐19, coronavirus Disease 2019; IRR, incidence rate ratio; KD, Kawasaki disease.

**TABLE 5:** IRRs of KD during the pre‐SARS‐CoV‐2 vaccine period.

	Events, *n*	IRR (95% CI)
Analysis: Day 0 out of the risk period	74	
Control period	12	Reference
Buffer period: Days −30 to −4	2	0.80 (0.18–3.6)
Pre‐exposure period: Days −3 to 0	20	55 (26–115)
Risk period: first month (Days 1 to 30)	24	8.7 (4.2–18)
Risk period: second month (Days 31 to 60)	12	4.3 (1.8–10)
Risk period: third month (Days 61 to 90)	4	1.4 (0.43–4.4)
Analysis: Day 0 in the risk period	74	
Control period	12	Reference
Buffer period: Days −30 to −4	2	0.78 (0.17–3.5)
Pre‐exposure period: Days −3 to −1	1	3.7 (0.47–29)
Risk period: first month (Days 0 to 30)	43	816 (8.0–32)
Risk period: second month (Days 31 to 60)	12	4.9 (2.0–12)
Risk period: third month (Days 61 to 90)	4	1.5 (0.48–4.9)

Abbreviations: CI, confidence interval; IRR, incidence rate ratio; KD, Kawasaki disease; SARS‐CoV‐2, severe acute respiratory syndrome coronavirus 2.

## 4 Discussion

In this study, we investigated the incidence of KD using data of 77,159 pediatric patients with COVID‐19 from a Japanese inpatient and outpatient database. Our results using nested case–control and SCCS design showed a significant increase in the risk of KD during the first and second months after COVID‐19 infection.

Several studies indicated the relationship between COVID‐19 and KD [10, 20] and, in our study, the odds ratio and incidence rate ratio of KD increased 2 months after COVID‐19 diagnosis. These results matched the timing of KD onset following SARS‐CoV‐2 exposure reported in the previous study [10]. Several factors may explain these findings. First, COVID‐19 may induce immune dysfunction relating to KD. Prior studies have indicated that T cell and B cell dysfunctions were associated with KD etiology [21, 22, 23], and SARS‐CoV‐2 infection can impair immune function [24, 25]. Therefore, T‐cell abnormalities triggered by SARS‐CoV‐2 infection may be associated with KD onset.

Second, changes in the gut microbiota associated with SARS‐CoV‐2 infection may have influenced KD. Dysbiosis within the gut microbiome is associated with KD [26], and COVID‐19 is known to induce dysbiosis in gut microbiota [27]. Therefore, COVID‐19‐induced gut microbial changes may lead to KD development.

In our study, the IRR increased during the pre‐exposure period and is possibly influenced by a delay between symptom onset and diagnosis. The date of diagnosis could be identified and was defined as Day 0 in this study. However, a duration of several days before Day 0 may be included in the risk period because a diagnosis of COVID‐19 may be delayed for several days following symptom onset [28]. Therefore, the observed increase in risk during the pre‐exposure period may be attributable to the effect of this diagnostic delay.

Consistent with previous studies [29], our data showed a sharp peak of events (KD) on Day 0 that may be attributed to the possibility that hospital visits or hospitalizations for the management of KD can trigger SARS‐CoV‐2 testing during routine screening on patient admission, with resultant incidental diagnoses of COVID‐19 that potentially inflate the observed association. To address this potential bias, the exclusion of Day 0 from the risk period is a reasonable approach. However, COVID‐19 may contribute to the peak on Day 0, because the onset of COVID‐19 can precede its diagnosis. Therefore, excluding Day 0 from the risk period may lead to an underestimation of the association between COVID‐19 and KD. In our study, elevated IRRs of KD were observed during the 3 months following COVID‐19 diagnosis in analyses that both included and excluded Day 0 from the risk period. As both analyses showed similar increases in IRRs, the findings in this study can be considered robust.

This study has several strengths. First, to our knowledge, the present study represents the first study that uses a nested case–control study and SCCS method for investigating the association between COVID‐19 and KD. Second, our inpatient‐and‐outpatient database provided a sufficient sample size and detailed patient background information to enable an assessment of the association between COVID‐19 and KD. Third, in addition to conducting a nested case–control study, we performed SCCS analysis. Thus, the nested case–control design reduces confounding by selecting controls from the same cohort, and the SCCS adjusts for time‐varying confounders. In this study, both analyses yielded similar results, which enhance the robustness of our results.

This study has some limitations. First, we were unable to investigate the relationship between COVID‐19 severity and KD. Second, the diagnostic accuracy of KD in our database might be uncertain. The cases with KD were identified based on the recorded ICD‐10 code of M303; on the other hand, the database did not include the clinical diagnostic process. Third, it has been reported that COVID‐19‐related Kawasaki‐like disease and multisystem inflammatory syndrome in children (MIS‐C) show clinical presentations similar to KD [30, 31], and our database did not know whether these diseases were distinguished from KD. However, MIS‐C is assigned a different ICD‐10 code than KD in the Japanese database, and KD and MIS‐C may have been distinguished in our database. Fourth, this study did not investigate the association between infectious diseases, except COVID‐19 and KD. Fifth, we were unable to investigate information on vaccination status. An elevated IRR for KD was observed in the analysis of the entire patient cohort, as in the subgroup analysis of pre‐vaccination. This finding may be related to the low vaccination coverage of SARS‐CoV‐2 among children [32].

In conclusion, the present study, using a Japanese inpatient and outpatient database, found that KD is associated with SARS‐CoV‐2 infection. We suggest that children may need appropriate infection controls and vaccination against SARS‐CoV‐2.

## Author Contributions

Tomoki Mizuno and Jun Suzuki had full access to all study data and accept responsibility for data integrity and the accuracy of the data analysis. Tomoki Mizuno, Jun Suzuki, Shota Takahashi, Haruka Imai, and Shiro Endo contributed to the conception and design of this study; all authors contributed to the acquisition, analysis, or interpretation of data; Tomoki Mizuno drafted the manuscript; Tomoki Mizuno, Jun Suzuki, and Tomohiro Akaba conducted statistical analysis; Jun Suzuki obtained funding; Tomoki Mizuno, Jun Suzuki, Shota Takahashi, Haruka Imai, and Hideya Itagaki provided administrative, technical, or material support; Tomohiro Akaba, Makiko Yoshida, and Shiro Endo supervised the whole study process. All authors read and approved the final manuscript.

## Funding

This work was supported by Grants‐in‐Aid for Scientific Research JSPS KAKENHI (grant number 23K16288).

## Ethics Statement

The study was approved by the Institutional Review Board of Tohoku Medical and Pharmaceutical University (Approval Number: 2023‐025). The requirement for informed consent was waived owing to the use of anonymized data.

## Consent

The requirement for informed consent was waived owing to the use of anonymized.

## Conflicts of Interest

The authors declare no conflicts of interest.

## Supporting information


**Table S1:** Procedure codes of the treatment for Kawasaki disease.

## Data Availability

Due to ethical considerations, the data cannot be publicly shared as it contains patient information. However, the study data can be made available to interested researchers upon reasonable request to the corresponding author, subject to ethical approval.
