# Supplementary material for: Ascertaining the Kawasaki Disease–Coronavirus Disease 2019 Linkage in a Japanese Administrative Claims Database
Source: Pediatr Int. 2026 Apr 28;68(1):e70420. doi: 10.1111/ped.70420 (PMC13123303; doi:10.1111/ped.70420)
Supplement: Supplementary file 1 — Table S1: Procedure codes of the treatment for Kawasaki disease. [file PED-68-e70420-s001.docx]

**Supplemental Table 1. Procedure codes of the treatment for Kawasaki disease**

| **Treatment** | **Procedure code** |
| --- | --- |
| Immunoglobulin | 621153301, 621152901, 621450001, 621151301, 621151701, 621449901, 621159901, 622534501, 622235601, 622534801, 622534701, 622534401, 622534601, 621160201, 621160501, 621490001, 621758002, 622192202, 622523501, 622192302, 621758102, 621758202 |
| Aspirin | 621419201, 621375001, 621374901, 621419401, 621374801, 622897400, 620000484, 620072715, 610443053, 620007816, 621525202, 622897300, 621675501, 622258001, 621676504, 611140850, 611140798, 620008577, 620072734 |
